# Sinus Lift-Before-Extraction Approach: Our Experience With a Strategic Approach to Implant Site Development

**DOI:** 10.7759/cureus.105584

**Published:** 2026-03-21

**Authors:** Fares Kablan, Amjad Shhadeh, Shadi Daoud, Mervat Khoury Absawi, Iris Slutzky-Goldberg, Samer Srouji

**Affiliations:** 1 Department of Oral and Maxillofacial Surgery, Galilee College of Dental Sciences, Galilee Medical Center, Nahariya, ISR; 2 The Azrieli Faculty of Medicine, Bar-Ilan University, Safed, ISR; 3 Department of Pediatric Dentistry, Galilee College of Dental Sciences, Galilee Medical Center, Nahariya, ISR; 4 Department of Endodontics, Galilee College of Dental Sciences, Galilee Medical Center, Nahariya, ISR

**Keywords:** dental implants, fixed prosthesis, hopeless teeth, maxillary sinus augmentation, posterior maxilla

## Abstract

Objectives: To present a sinus lift-before-extraction staged approach for maxillary sinus augmentation in patients with posterior maxillary teeth of poor or hopeless prognosis. In this protocol, lateral sinus augmentation is performed before tooth extraction and implant placement to increase available bone height for subsequent rehabilitation.

Method and materials: This retrospective case series included 16 posterior maxillary sites in 13 patients treated over an eight-year period. All cases required lateral window sinus augmentation in regions with compromised teeth planned for future extraction and implant-supported rehabilitation. Preoperative cone-beam computed tomography demonstrated a mean residual bone height of 4 mm. Following augmentation, the non-restorable teeth were temporarily retained during healing to support provisional restorations and maintain function and esthetics. Tooth extraction and implant placement were then performed as a second-stage procedure.

Results: Between 2014 and 2022, sinus augmentation was performed as the initial stage in 13 patients (43-69 years). Healing was uneventful, and morbidity was minimal. At the second stage, the compromised teeth were extracted, and 39 implants were placed in the augmented sites. All implants achieved osseointegration and were restored with fixed implant-supported prostheses. The mean follow-up was 60 months. Patient-reported satisfaction was favorable.

Conclusion: A sinus lift-before-extraction staged approach appears to be a feasible approach with favorable clinical, radiographic, and patient-reported outcomes in selected posterior maxillary sites associated with hopeless teeth. Temporarily retaining the teeth during healing may help preserve interim function and esthetics while enabling implant placement after augmentation.

## Introduction

The edentulous posterior maxilla frequently presents with insufficient bone volume due to a combination of maxillary sinus pneumatization and alveolar ridge resorption. Since Tatum's pioneering report in 1986 [1], sinus floor augmentation has become a well-established procedure for vertical bone augmentation in the posterior maxilla, most commonly performed either simultaneously with implant placement or as a staged approach after tooth extraction and healing [2-4]. In contrast, performing sinus augmentation prior to the extraction of compromised posterior teeth has been only sparsely described, despite its potential advantages in selected cases.

Management of severely compromised teeth in the posterior maxilla poses a unique clinical challenge when planning for sinus augmentation and implant placement. Such teeth are often affected by advanced periodontal disease, vertical root fractures, internal or external resorption, or extensive caries, rendering them non-restorable [5,6]. The designation of these teeth as "hopeless" is typically based on a comprehensive clinical and radiographic evaluation, including periodontal, restorative, and endodontic considerations.

Treatment strategies in these scenarios can be broadly categorized into three main protocols: (1) simultaneous sinus augmentation with immediate implant placement, with or without immediate functional loading; (2) extraction followed by immediate-delayed implant placement with concurrent sinus grafting (typically within six to eight weeks of extraction); (3) delayed approach, where sinus augmentation and implant placement are performed approximately four months after extraction, often following socket preservation procedures [7]. Immediate loading may be considered in selected cases when adequate primary implant stability is achieved, commonly reflected by insertion torque of at least 30 Ncm and/or implant stability quotient (ISQ) values of at least 60, as suggested in the literature [8].

Treatment planning should be individualized, taking into account patient-specific factors such as overall health, cost considerations, treatment timeline, and preferences of both the patient and dental team. Interestingly, even hopeless teeth may serve a transitional role in treatment. Retaining these teeth temporarily prior to extraction and implant placement can ease the patient’s adaptation process, particularly compared to abrupt changes like immediate partial or complete dentures. This staged approach may help reduce the edentulous phase and maintain interim function during the transition to implant-supported prostheses [9]. In the present study, these patient-centered aspects were assessed descriptively using a structured questionnaire addressing postoperative discomfort, functional limitations during healing, and overall satisfaction.

However, the literature provides limited clinical descriptions of sinus augmentation performed prior to the extraction of compromised posterior maxillary teeth, and the indications, practical advantages, and clinical outcomes of this sequencing strategy remain insufficiently characterized. In this paper, we present our clinical experience with an alternative staged approach: maxillary sinus augmentation performed prior to the extraction of compromised posterior maxillary teeth. This method may offer theoretical advantages, including preservation of anatomical landmarks and facilitation of prosthetically driven planning. In the present case series, the evaluated outcomes included radiographic bone gain prior to implant placement, implant survival/success, postoperative complications, and patient-reported outcomes. This retrospective case series reports the clinical and radiographic outcomes of eligible treated cases, including radiographic bone gain, implant survival/success, postoperative complications, and patient-reported outcomes; selected case examples are presented only to illustrate the surgical protocol.

## Materials and methods

Treatment protocol

This study is a retrospective, non-consecutive case series conducted at the Department of Oral and Maxillofacial Surgery, Galilee Medical Center, between 2014 and 2022. Cases were identified retrospectively from treated patients who met the predefined eligibility criteria and had sufficiently complete clinical, radiographic, and follow-up documentation for analysis.

The sinus lift-before-extraction approach was employed for the reconstruction of the posterior maxilla in cases involving teeth with a poor or hopeless prognosis requiring future replacement with dental implants. Each patient underwent a thorough preoperative assessment, including medical history review, clinical examination, and cone-beam computed tomography (CBCT) imaging to evaluate the available residual bone height (RBH) in the posterior maxilla. Sinus health was assessed based on clinical symptoms and CBCT findings; patients with signs or symptoms of active sinusitis or radiographic sinus pathology requiring otolaryngologic evaluation were excluded, and ENT consultation was obtained when indicated. RBH was measured preoperatively on cross-sectional CBCT images oriented perpendicular to the alveolar crest at the planned implant site, as the vertical distance from the alveolar crest to the sinus floor, and this method was applied consistently across cases. At the four-month evaluation, RBH was assessed primarily with CBCT; in three patients where CBCT was not available, standardized periapical radiographs obtained with a reproducible paralleling technique were used, with dimensional calibration based on the known implant length. These non-CBCT assessments were used to support clinical decision-making regarding readiness for implant placement.

Teeth deemed non-restorable were identified based on clinical and radiographic parameters, such as advanced periodontal breakdown, endodontically treated teeth considered non-restorable because of extensive coronal destruction, unfavorable remaining tooth structure for predictable restoration, or root fracture, but without clinical or radiographic signs of active infection. A staged surgical protocol was followed. In the first stage, a lateral window sinus augmentation was performed while retaining the compromised teeth to allow for interim restorations, thereby preserving function and esthetics during the healing period. After a planned healing period of approximately four months, a second-stage surgery was performed, during which the hopeless teeth were extracted, and dental implants were placed, with the timing confirmed by radiographic evidence of bone gain and clinical assessment of readiness for implant placement. Implant loading, either immediate or delayed, was determined based on primary implant stability, generally reflected by insertion torque of at least 30 Ncm and/or ISQ values of at least 60. In cases where delayed loading was selected, final prosthetic rehabilitation was completed within three to four months following implant placement.

Patient-reported outcomes were collected using a study-specific, non-validated questionnaire (Appendices) composed of a stage-specific postoperative survey and a final treatment satisfaction survey. The questionnaire assessed postoperative discomfort, swelling, eating/speaking comfort during healing, satisfaction with care, esthetic and functional satisfaction, perceived value of treatment, comfort with the staged timeline, and overall treatment acceptance. Responses were recorded primarily on a five-point ordinal scale and analyzed descriptively. The stage-specific questionnaire was completed during the postoperative healing phase, whereas the final treatment satisfaction questionnaire was completed after definitive prosthetic rehabilitation. All patients signed written informed consent for treatment. Clinical photographs and radiographic images included in the manuscript were fully de-identified.

Inclusion criteria were patients presenting with posterior maxillary teeth of hopeless prognosis adjacent to the planned sinus augmentation site, insufficient RBH generally ≤5 mm, for predictable implant placement without sinus augmentation, and a treatment plan for implant-supported rehabilitation.

Exclusion criteria included active sinus disease/sinusitis, radiographic sinus pathology requiring ENT management, uncontrolled periodontitis, and any odontogenic infection in the surgical field, including symptomatic teeth, acute apical periodontitis, draining sinus tract, swelling, suppuration, and teeth with radiographic periapical radiolucency or other signs of endodontic-periodontal lesions in proximity to the sinus floor. Teeth with untreated deep caries, non-contained coronal destruction with suspected pulpal infection, or persistent endodontic infection were also excluded unless infection was definitively resolved prior to augmentation. Systemic exclusion criteria included American Society of Anesthesiologists (ASA) class III-IV, immunosuppression, antiresorptive therapy (e.g., bisphosphonates), history of head and neck radiotherapy, and pregnancy. Smoking status was documented for all patients, but was not an exclusion criterion.

Teeth were classified as hopeless based on combined clinical and radiographic evaluation (e.g., severe periodontal attachment loss with grade III mobility, advanced furcation involvement, poor crown-to-root ratio compromising restorability, vertical/extensive root fracture, or non-restorable coronal destruction). Classification was performed by the treating surgeon based on these predefined clinical and radiographic considerations. Importantly, only teeth without clinical or radiographic evidence of active infection were retained temporarily during the graft-healing phase. Accordingly, no tooth retained during the graft-healing phase demonstrated swelling, suppuration, sinus tract, or other clinical signs of odontogenic infection, and preoperative CBCT showed no periapical radiolucency or endodontic-periodontal lesion adjacent to the sinus floor. During the healing phase, retained teeth were monitored at monthly follow-up visits by clinical examination, with interim radiographic assessment performed when clinically indicated.

Implant success was defined according to standard clinical and radiographic criteria: absence of pain, infection, or mobility; absence of continuous peri-implant radiolucency on radiographs; and the presence of functional loading with a stable prosthetic restoration. Survival was defined as the implant remaining in situ, regardless of prosthetic or biological complications. Success and survival were assessed at the time of prosthetic loading and at the most recent documented follow-up visit.

As this was a retrospective case series spanning 2014-2022, the length of follow-up varied according to the year of treatment, with earlier patients naturally having longer observation and more recent patients shorter. All patients were followed until their most recent documented clinical and prosthetic evaluation. Complications were identified retrospectively from the clinical and radiographic records, including postoperative infection, wound dehiscence, inflammatory events related to retained teeth, sinus-related symptoms, implant failure, and biological or mechanical prosthetic complications.

Sinus lift surgery

At the first surgical appointment, prophylactic antibiotic therapy was initiated 24 hours prior to surgery and continued for seven days postoperatively (total course: eight days). Patients received either amoxicillin-clavulanic acid (875/125 mg, twice daily) or, in cases of penicillin allergy, clindamycin 300 mg (three times daily). Local anesthesia was administered using 2% lidocaine with 1:100,000 epinephrine (3M ESPE, St. Paul, MN).

A full-thickness envelope flap was elevated in the posterior maxilla to expose the lateral sinus wall. A lateral window (antrostomy), sized according to the extent of the planned augmentation and local anatomy, was created using a round diamond bur operating at 1000 rpm under copious sterile saline irrigation. The Schneiderian membrane was carefully elevated using sinus curettes superior to the roots of the adjacent teeth. No Schneiderian membrane perforations were observed intraoperatively. The sinus cavity was then grafted with an allogenic particulate bone substitute (Raptos, Citagenix, Laval, Quebec, Canada; particle size 250-1000 µm), which was used consistently across all cases. No barrier membrane was placed over the lateral window. The flap was repositioned and sutured for primary closure.

Postoperative care

Postoperative antibiotics were continued as per the preoperative regimen. Additionally, patients were prescribed a 0.12% chlorhexidine gluconate mouth rinse to be used twice daily for one week. By postoperative day 10, mucosal healing was typically considered complete based on clinical soft-tissue closure without wound dehiscence, membrane exposure, or signs of local infection, and sutures were removed. Patients attended monthly follow-up appointments to assess healing progression, postoperative complications, and adverse events clinically, with radiographic evaluation performed when indicated and documented in the follow-up records. Retained teeth were also assessed at each visit for pain, increasing mobility, periodontal deterioration, and any signs of endodontic or odontogenic exacerbation.

Stage 2: Tooth extraction and implant placement

Four months after sinus augmentation, periapical radiographs or a CBCT scan were performed to evaluate bone regeneration and confirm the suitability of the grafted site for implant placement. Readiness for implant placement was determined by radiographic evidence of sufficient vertical bone gain to allow placement of the planned implant in the correct prosthetic position, with adequate radiographic mineralization of the grafted area, complemented by clinical assessment of bone quality during osteotomy preparation. The second surgical procedure included the extraction of the compromised maxillary teeth and the placement of dental implants.

A total of 39 implants (diameter: 3.75 or 4.2 mm; length: 11-13 mm) were placed across the 16 augmented posterior maxillary sites, with multiple implants placed within several augmented regions according to the prosthetic plan. Implant placement was performed either with surgical templates when prosthetically indicated or freehand according to the clinical situation. After an additional healing period of three to four months, definitive prosthetic rehabilitation was completed with fixed implant-supported restorations, achieving stable functional and esthetic outcomes.

## Results

Case illustration 1

**Figure 1:**
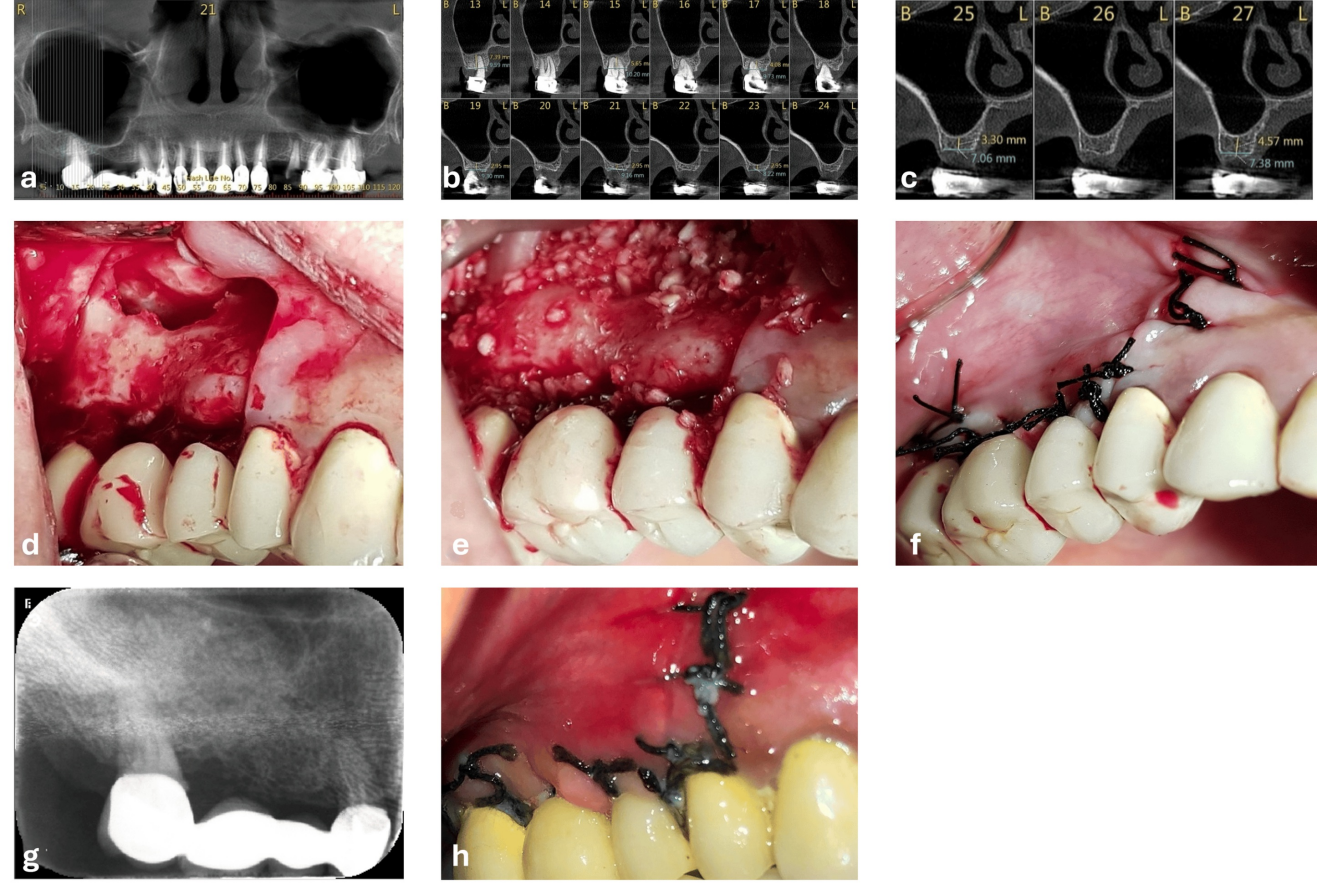
Clinical and radiographic presentation of case 1. (a-c) Preoperative cone-beam computed tomography images showing vertical bone deficiency at the right maxillary sinus region. The first premolar and second molar served as abutments for a fixed prosthesis, while the second premolar and first molar were missing. (d) Access to the maxillary sinus following envelope flap elevation. (e) Sinus augmentation was performed through the lateral window approach. (f) Primary flap closure achieved without tension. (g) Postoperative radiograph showing the augmented sinus; the existing fixed prosthesis was retained temporarily during the healing phase. (h) Clinical view at 10-day follow-up demonstrating intact mucosa and absence of wound dehiscence.

A 57-year-old female patient was referred for maxillary sinus augmentation and implant placement in the right posterior maxilla to replace teeth with a hopeless prognosis. The patient was considered systemically healthy, with no medical conditions, but reported the daily use of low-dose acetylsalicylic acid (100 mg/day) as preventive therapy only, together with calcium and vitamin D supplementation. She reported a smoking habit of approximately 10 cigarettes per day. She was counseled preoperatively regarding smoking reduction/cessation and the potential adverse effects of smoking on healing, and the low-dose acetylsalicylic acid therapy was not discontinued perioperatively because the planned procedure was performed under local hemostatic control. Clinical examination revealed detachment of an old fixed dental prosthesis extending from the maxillary right first premolar to the second molar. Both the first premolar and second molar, which served as abutments, showed significant periodontal breakdown with grade III mobility. Deep subgingival secondary caries was present on the first premolar. Despite the poor restorative prognosis, the tooth was asymptomatic, with no swelling/suppuration/sinus tract, and CBCT showed no periapical radiolucency adjacent to the sinus floor. During the four-month healing period, no active endodontic or periodontal intervention was required; the patient was maintained with routine clinical follow-up and oral hygiene instructions. The maxillary right second premolar and first molar were missing (Figure 1A).

CBCT demonstrated a residual alveolar bone height ranging from 3 to 6 mm in the edentulous posterior maxilla (Figures 1B, 1C). A staged treatment approach involving a sinus lift prior to tooth extraction was proposed. This plan was selected based on two considerations: (1) the patient’s refusal to wear a removable partial denture during the healing period, and (2) the surgeon’s preference to avoid immediate implant placement and loading in conjunction with sinus augmentation because of the increased technical and biological demands in sites with limited RBH, including the risk of insufficient primary stability, membrane perforation, and compromised healing in smokers. The patient reported familiarity with the staged sinus lift-before-extraction approach and had specifically sought referral for this protocol.

Following the patient’s informed consent, the initial surgical procedure was performed. This involved the elevation of an envelope flap and a lateral window sinus augmentation (Figures 1D, 1E). Flap closure was achieved without tension (Figure 1F). The postoperative healing period was uneventful, with only mild swelling and minimal discomfort documented at the postoperative follow-up visits. Sutures were removed on postoperative day 10 (Figures 1G, 1H).

**Figure 2:**
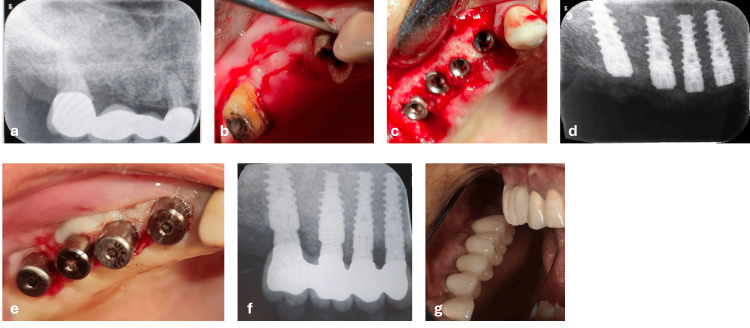
Second surgical stage and prosthetic rehabilitation of case 1. (a) Periapical radiograph obtained four months after sinus augmentation. (b) Extraction of hopeless teeth in the posterior maxilla. (c) Intraoperative view showing placement of four dental implants. (d) Postoperative radiograph confirming the placement of implants. (e) Healing caps placement. (f, g) Demonstrate the final prosthetic rehabilitation with a fixed prosthesis supported by four implants, restoring the posterior maxillary dentition.

At four months post-sinus lift (Figure 2A), the second surgical stage was performed. The maxillary right first premolar and second molar were extracted (Figure 2B), and four implants (length: 11.5-13 mm; diameter: 3.7-4.2 mm) were placed in the augmented area (Figures 2C, 2D). Following two weeks with healing caps (Figure 2E), prosthetic rehabilitation proceeded according to the delayed loading protocol described in the Methods, and definitive fixed prosthetic rehabilitation was completed four months after implant placement (Figures 2F, 2G). The total treatment duration was eight months. According to the final treatment satisfaction questionnaire completed after definitive prosthetic rehabilitation, the patient rated both the functional and esthetic outcomes as “very satisfied.”

Case illustration 2

**Figure 3:**
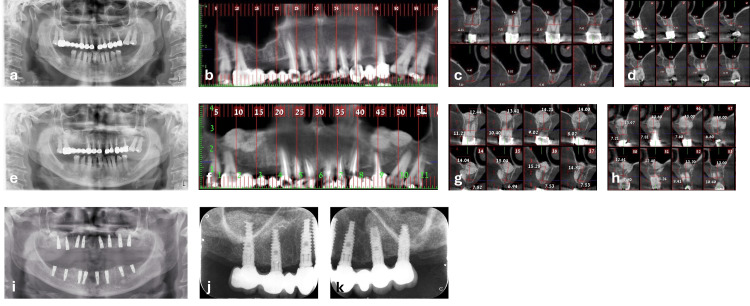
Preoperative and postoperative radiographic views of case 2. (a) Preoperative panoramic radiograph showing hopeless posterior maxillary teeth bilaterally and vertical bone deficiency. (b-d) Preoperative cone-beam computed tomography (CBCT) images confirming the vertical bone deficiency at the posterior maxilla. (e) Postoperative panoramic radiograph following bilateral sinus augmentation, performed prior to tooth extraction. (f-h) Postoperative CBCT images demonstrating new bone formation following bilateral sinus lift procedures. (i) Panoramic radiograph after the second surgical stage, including tooth extractions and implant placement. (j-k) Follow-up radiographs at 10.5 years postoperatively.

A 50-year-old medically healthy female patient (ASA grade I, with no relevant systemic comorbidities identified on preoperative medical history screening) was referred for bilateral maxillary sinus augmentation and the placement of eight dental implants in the maxilla. Clinical and radiographic evaluations revealed failing fixed prostheses extending from the maxillary right second molar to the left second premolar. The prostheses included pontics replacing the right first premolar, second premolar, and first molar, as well as the left first premolar. The left first molar had been extracted during childhood. The remaining abutment teeth exhibited extensive secondary caries and advanced periodontal bone loss (Figure 3A).

CBCT evaluation revealed RBH of 4-7 mm in the right posterior maxilla and 4-6 mm in the left posterior maxilla (Figures 3B-3D). The initial treatment plan involved the extraction of the compromised teeth, socket preservation, and temporary rehabilitation with a complete maxillary denture. After four to five months of healing, a second surgical stage would be performed, consisting of bilateral sinus augmentation and placement of eight implants. A fixed full-arch implant-supported prosthesis would then be delivered following successful osseointegration. However, given the limited bilateral RBH, the absence of active odontogenic infection, and the prosthetic goal of fixed full-arch rehabilitation, a sinus lift-before-extraction strategy was considered a suitable alternative to avoid a prolonged edentulous phase. This approach also aligned with the patient’s wish to maintain interim function and appearance before her daughter’s wedding, scheduled in three months; she had previously consulted another surgeon who suggested this strategy and referred her to our department specifically for this approach.

After discussing the alternative treatment plan and obtaining informed consent, the decision was made to proceed with a sinus lift prior to extractions.

The first surgery was performed under local anesthesia and included bilateral lateral window sinus augmentation using an allogenic particulate bone substitute (Figure 3E). The postoperative course was uneventful, with smooth healing observed during regular follow-ups. Due to patient scheduling considerations, stage 2 was performed eight months postoperatively. At that time, a CBCT scan confirmed sufficient vertical bone gain and radiographic mineralization in the grafted area to allow implant placement in the planned prosthetic position in both posterior maxillary regions (Figures 3F-3H). The second stage was then performed. All remaining maxillary teeth were extracted, and eight dental implants (3.75-4.2 mm diameter, 13 mm length) were placed, four in the anterior maxilla (premaxilla), two in the right posterior region, and two in the left posterior region. Additionally, socket preservation was performed in the anterior maxilla (central incisor region) to optimize future esthetics. Concurrent mandibular implant treatment was also carried out at this stage (Figure 3I). Accordingly, patient-reported postoperative discomfort at this time point may have reflected the combined surgical experience rather than the maxillary procedure alone.

Following an additional four months of healing, the patient underwent final full-mouth rehabilitation with implant-supported fixed prostheses. The treatment resulted in high patient-reported esthetic and functional satisfaction, with no recorded prosthetic complications during follow-up. The patient has been followed for 126 months (Figures 3J, 3K), during which the implants remained functional and in situ, fulfilling the predefined survival/success criteria, with no recorded biological or prosthetic complications. Based on her responses to the satisfaction questionnaire, she expressed a high level of satisfaction with the results and appreciated that the modified treatment approach enabled her to initiate therapy without compromising her plans for her daughter’s wedding.

Thirteen subjects (nine females and four males; mean age = 52 years, range = 43-69) were included in this retrospective, non-consecutive case series conducted between 2014 and 2022 (Table 1). Follow-up ranged from 2.5 to 10.5 years. The healing process was uneventful, with no postoperative infections, wound dehiscence, inflammatory complications related to retention of hopeless teeth, or clinical/radiographic signs of impaired sinus function or ostiomeatal obstruction during follow-up. Patients reported only mild swelling and discomfort, and they were able to continue eating and chewing as they did before surgery. At the suture removal appointment, 10 days postoperatively, the surgical sites exhibited excellent healing without any signs of wound dehiscence. Four months after the procedure, CBCT imaging confirmed adequate bone height for implant placement.

**Table 1:** Demographic and clinical data of the study patients. M: male; F: female; RT: right; LT: left; PM: premolar; M: molar; RBH: residual bone height.

Patient No.	Age	Gender	Site	Involved teeth	Preoperative RBH (mm)	Smoking	Surgery date	Follow-up (years)
1	50	F	Bilateral	RT: 2nd & 3rd M. LT: 2nd PM, 2nd M	5-7, 4-6	No	09/2014	10.5
2	44	F	RT	2nd PM, 1st M	3-6	No	02/2015	10
3	54	M	LT	2nd PM, 2nd M	4-6	Yes	05/2015	10
4	45	F	Bilateral	RT: 2nd & 1st M. LT: 2nd & 1st M	3-5, 3-5	No	03/2020	4
5	59	F	RT	1st PM, 2nd M	3-4	Yes	04/2021	4
6	61	M	LT	2nd PM, 2nd M	1-5.0	Yes	04/2021	4.4
7	57	F	LT	1st PM, 2nd M	4.5-5.5	­Yes	07/2021	4.8
8	52	F	RT	1st PM, 2nd M	2-4	No	11/2021	4.4
9	45	M	LT	2nd PM, 1st M	2-5, 1-3	No	12/2021	4.5
10	52	F	RT	1st PM, 2nd M	4-7	No	07/2022	2.8
11	43	F	LT	1st M, 2nd M	3-6	No	08/2022	2.8
12	56	F	Bilateral	RT: 2nd PM, 2nd M. LT: 2nd PM, 2nd M	1-4, 1-3	No	11/2022	2.5
13	46	M	RT	1st PM, 1st M	2-4	No	12/2022	2.5

A total of 39 implants of appropriate length (11-13 mm) and diameter (3.75-4.2 mm) were successfully placed in 16 sites on the posterior maxilla. The treated sites healed well, and all implants fulfilled the predefined success criteria, demonstrating stable osseointegration. No biological complications (including peri-implant mucositis, peri-implantitis, or implant loss) or mechanical complications (including screw loosening, prosthetic fracture, or loss of prosthesis stability) were recorded during follow-up. Of the 13 patients, four were smokers, and nine were non-smokers. All implants achieved osseointegration in this limited cohort; however, the cohort size was too small to permit meaningful comparison between smokers and non-smokers or to assess smoking as a potential confounder. Final rehabilitation was completed with fixed prostheses over the implants.

Patient-reported outcomes, assessed using a five-point ordinal questionnaire during the postoperative healing phase and after definitive prosthetic rehabilitation, indicated an overall favorable response to the sinus lift-before-extraction approach. During healing, pain/discomfort and swelling were generally rated in the mild-to-moderate range, while satisfaction with care was high, with 11 of 13 patients (84.6%) assigning the highest score. After definitive prosthetic rehabilitation, esthetic satisfaction was rated at the highest level by 11 of 13 patients (84.6%), and functional comfort by 12 of 13 patients (92.3%). Overall satisfaction was reported as “very satisfied” by 10 of 13 patients (76.9%) and “satisfied” by three of 13 patients (23.1%). In addition, 11 of 13 patients (84.6%) stated that they would recommend the treatment to others with similar conditions, and 12 of 13 (92.3%) indicated that they would undergo the treatment again. These findings suggest that the staged approach was well accepted and contributed positively to the overall patient experience.

## Discussion

The sinus lift-before-extraction approach represents a modification of conventional sinus augmentation protocols for patients with hopeless posterior maxillary teeth and insufficient residual bone height. In the present series, the favorable postoperative course and positive patient-reported outcomes support its potential patient-centered benefits, whereas reduced surgical complexity should be regarded as a theoretical procedural advantage rather than a directly measured outcome.

Grafting the maxillary sinus floor is one of the most commonly performed surgical procedures to increase alveolar bone height for placement of endosseous dental implants in the posterior maxilla since its first publication by Tatum in 1986 [1]. The success of this approach depends on several factors, including the selected surgical approach and the timing of implant placement, whether simultaneous or delayed. In a systematic review, Romero-Millán et al. reported that implants placed in the posterior maxilla following open sinus lift showed clinical outcomes comparable to those of implants placed in native bone, although sinus lift procedures were associated with a higher incidence of surgical complications [10].

Traditionally, in cases of significant sinus pneumatization with a residual alveolar bone height of less than 5 mm, lateral sinus augmentation has been considered necessary [11]. When the available bone measures between 1 and 3 mm, a staged approach was recommended [12]. However, some surgeons now perform one-stage surgery even when the residual alveolar bone height is less than 4 mm. In a prospective study by Cha et al., implants placed simultaneously with sinus lift in sites with RBH <4 mm showed high cumulative survival and success rates, without a statistically significant difference in success compared with sites with RBH >5 mm, when primary stability was achieved [13].

The presence of non-restorable teeth often requires extraction, followed by implant placement to support a fixed prosthesis. In similar cases, several treatment approaches can be considered: a one-stage approach involves extracting the teeth, performing a sinus lift, and placing implants in a single procedure. While this can shorten the overall treatment timeline, it presents several intraoperative challenges. These include the need for a wide flap elevation, reduced visibility due to concurrent extractions and bleeding, increased risk of sinus membrane perforations, and limitations in implant placement, as implants can only be positioned where sufficient residual bone is available, rather than at the optimal prosthetic location.

One of the most significant challenges with the simultaneous approach is achieving primary closure over the extraction sockets, particularly in the molar region. This often necessitates extensive flap elevation with wide releasing incisions, periosteal releasing cuts, and, in some cases, soft tissue grafting to ensure proper closure. Due to its invasiveness, this approach may be associated with a higher risk of postoperative morbidity and surgical complications, particularly those related to flap advancement and lateral sinus augmentation. A systematic review identified sinus membrane perforation, wound dehiscence, graft exposure/failure, and sinusitis as the most common complications requiring intervention after lateral sinus floor elevation [14]. Patients may express anxiety or concern regarding sinus lift surgery. A recent study found that while patients generally reported high satisfaction with dental implant treatments, factors such as implant failure, mechanical complications, and the necessity for sinus augmentation negatively impacted satisfaction levels [15]. Staged surgery involves extracting non-restorable teeth first, followed by a delayed sinus lift and implant placement. Implant placement can be performed as immediate-early, within four to eight weeks [16], or as delayed, typically at least four months after extraction [17]. Additionally, the staged approach includes a socket augmentation with delayed sinus lift, and implant placement can be performed. In this approach, hopeless teeth are extracted, and the sockets are augmented. The sinus lift and implant placement were performed after four to five months of healing. A key advantage of both staged approaches is that they allow sufficient healing time for the extraction sites, ensuring good soft tissue primary closure over the augmented sinus. However, these approaches can extend the overall treatment duration and increase costs.

We have employed the sinus lift-before-extraction approach in the posterior maxilla. This approach is indicated in cases of maxillary sinus pneumatization with insufficient residual bone requiring lateral sinus augmentation, combined with the presence of non-restorable teeth destined for replacement with implant-supported fixed prostheses. It has been reported that extending sinus floor augmentation beyond the edentulous area, apical to the adjacent teeth, has not been associated with increased postoperative complications in available clinical studies. In a clinical study of 65 patients, Beitlitum et al. reported minor membrane perforations in four cases, with no postsurgical graft contamination or periradicular changes during follow-up, and concluded that this technique may facilitate future implant placement while avoiding sinus reentry and extraction of proximal teeth [18].

Based on our experience, performing the sinus lift as a standalone procedure during the first stage, without concurrent tooth extractions or implant placement, may simplify the surgical workflow. Improved visibility and access to the lateral sinus wall can be achieved with an envelope flap around the retained teeth, facilitating rapid and precise window preparation. Separating sinus augmentation from extractions may also facilitate flap closure and stable graft coverage during healing [19,20]. In our cohort, no wound dehiscence or Schneiderian membrane perforations were recorded. Together with temporary preservation of hopeless teeth for interim function and esthetics, these features may have contributed to the favorable postoperative course and positive patient-reported outcomes observed in this series.

The author utilizes allogenic particulate bone substitutes as the sinus graft material within a treatment protocol planned for re-entry at approximately four months, thereby aiming to reduce the overall treatment period. The use of allografts in sinus lift procedures has been shown to be safe and effective [21]. In the present series, the second-stage procedure was generally scheduled after a planned healing period of approximately four months, with final timing confirmed by radiographic evidence of bone gain and clinical assessment of readiness for implant placement. In our experience with this approach, the total treatment duration ranges from approximately five months in cases of immediate implant loading to eight months in cases of delayed implant loading.

Regarding the treatment timeline, the staged nature of this approach appeared to be well accepted by patients. Overall, the patient-reported findings suggest that, beyond its clinical feasibility, this approach may contribute positively to the treatment experience by reducing the edentulous period and supporting functional and esthetic adaptation during rehabilitation.

While the sinus lift before tooth extraction approach offers notable advantages in patient adaptation, prosthetic planning, and anatomical preservation, this study has several limitations. The sample size was limited, and the absence of a control group prevents direct comparison with traditional protocols such as extraction prior to sinus augmentation. Although smoking status was recorded, the limited cohort size did not allow for a meaningful assessment of its potential confounding effect on treatment outcomes. The follow-up period, although sufficient for initial outcomes, does not provide insight into long-term implant survival and peri-implant bone stability. To validate the clinical utility of this approach, larger randomized controlled trials are needed comparing this approach with standard delayed and immediate placement protocols. The two illustrative cases presented in detail were chosen to demonstrate the surgical protocol step-by-step; they do not necessarily reflect the full demographic diversity of the study population. In addition, the use of mixed imaging modalities at the four-month assessment may have introduced measurement variability, as three sites were evaluated with calibrated periapical radiographs rather than CBCT. Finally, the retrospective design and the fact that all surgeries were performed by a single surgeon may introduce subjective bias and limit the generalizability of the findings.

## Conclusions

The sinus lift-before-extraction approach may represent a useful modification of the conventional sequence for selected cases in the atrophic posterior maxilla. Its main advantage is the temporary maintenance of compromised teeth during healing, which can support provisional restorations and help preserve function and esthetics until implant placement. However, predictable outcomes depend on careful case selection and meticulous preoperative planning, including assessment of residual bone height, periodontal/endodontic status, and infection control. Although implant-related, complication-related, and patient-reported outcomes were favorable in this series, further well-designed studies are required to confirm long-term effectiveness and define clear indications and limitations.

## Figures and Tables

**Figure 4:**
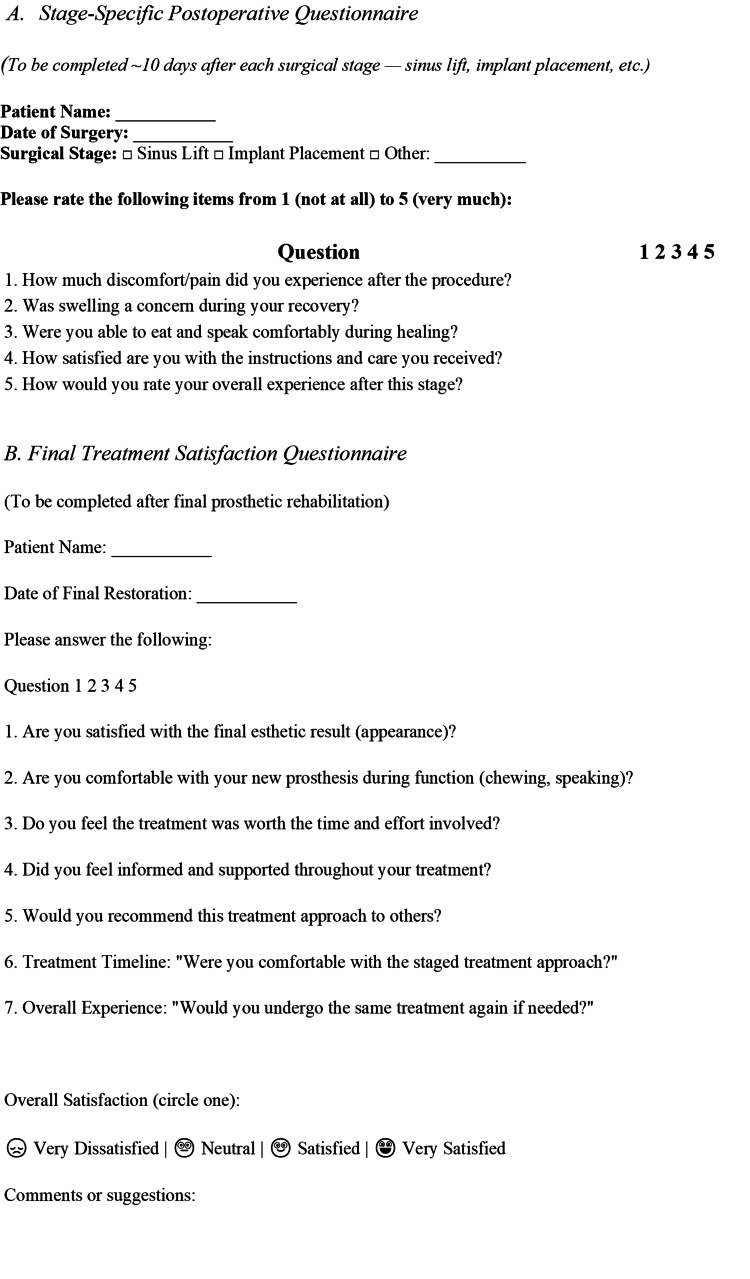
Satisfaction questionnaire.
